# Effects of Low Salinity on Adult Behavior and Larval Performance in the Intertidal Gastropod *Crepipatella peruviana* (Calyptraeidae)

**DOI:** 10.1371/journal.pone.0103820

**Published:** 2014-07-31

**Authors:** Jaime A. Montory, Jan A. Pechenik, Casey M. Diederich, Oscar R. Chaparro

**Affiliations:** 1 Instituto de Ciencias Marinas y Limnológicas, Universidad Austral de Chile, Valdivia, Chile; 2 Biology Department, Tufts University, Medford, Massachusetts, United States of America; 3 Programa de Doctorado en Ciencias Mención Ecología y Evolución, Universidad Austral de Chile, Valdivia, Chile; University of Hong Kong, Hong Kong

## Abstract

Shallow-water coastal areas suffer frequent reductions in salinity due to heavy rains, potentially stressing the organisms found there, particularly the early stages of development (including pelagic larvae). Individual adults and newly hatched larvae of the gastropod *Crepipatella peruviana* were exposed to different levels of salinity stress (32(control), 25, 20 or 15), to quantify the immediate effects of exposure to low salinities on adult and larval behavior and on the physiological performance of the larvae. For adults we recorded the threshold salinity that initiates brood chamber isolation. For larvae, we measured the impact of reduced salinity on velar surface area, velum activity, swimming velocity, clearance rate (CR), oxygen consumption (OCR), and mortality (LC50); we also documented the impact of salinity discontinuities on the vertical distribution of veliger larvae in the water column. The results indicate that adults will completely isolate themselves from the external environment by clamping firmly against the substrate at salinities ≤24. Moreover, the newly hatched larvae showed increased mortality at lower salinities, while survivors showed decreased velum activity, decreased exposed velum surface area, and decreased mean swimming velocity. The clearance rates and oxygen consumption rates of stressed larvae were significantly lower than those of control individuals. Finally, salinity discontinuities affected the vertical distribution of larvae in the water column. Although adults can protect their embryos from low salinity stress until hatching, salinities <24 clearly affect survival, physiology and behavior in early larval life, which will substantially affect the fitness of the species under declining ambient salinities.

## Introduction

Organisms that live intertidally, in shallow coastal waters, or near riverine or glacial outputs are naturally subject to drastic fluctuations in environmental conditions [1], [2], [3], [4], [5], [6], [7], including changes in salinity. In certain situations (e.g. during heavy rains), the water can reach almost freshwater conditions [8], [9], [10], [11], [12], imposing severe physiological challenges on many residents [13], [14], [15], [16], [17], [18], which may affect distribution, behavior and/or survival [17], [19], [20], [21], [22], [23], [24], [25], [26].

Exposure to environmental stress generally increases the vulnerability of individuals during early development [27], [28]. Adults routinely exposed to such salinity fluctuations often respond with escape behaviors [29] or by producing copious amounts of corporal mucous [30]. Also, shelled organisms may be able to isolate their tissues for long periods of time through valve or operculum closure [10], [11], [20], [31], [32], [33]. The intertidal gastropod *Crepipatella dilatata*, for example, will isolate its embryos from the external environment when salinities fall below 22 [33]. The present study determines whether adults of *Crepipatella peruviana* (formerly *Crepipatella fecunda* [34]) also exhibit this protective behavior, and if so, at what salinity the behavior is initiated. After hatching, however, the free-living larval stages may also be exposed to low-salinity stress, which can be particularly detrimental because they lack adequate physical protection [35] and because they no longer have the maternal physical protection that could otherwise potentially have isolated them from the stressful environmental conditions [33]. Not surprisingly, salinity stress can dramatically affect larval behavior and development [7], [26], [36], [37], [38], [39], [40], [41], increase developmental mortality [23], alter rates of oxygen consumption [42], [43], [44], [45], negatively impact swimming activity [28], [46] and feeding rates [7]. In addition, discontinuous salinities can notably alter the distribution of larvae in the water column [26], [46].

The protandous gastropod *Crepipatella peruviana* [47], [48] is a common inhabitant of intertidal and shallow subtidal areas of southern Chile. Like other members of the family Calypraeidae, females incubate their egg masses within the mantle cavity beneath the shell for about 4 weeks before releasing feeding veliger larvae into the plankton [50], [51] at shell lengths (SL) of approximately 350 µm [48]. The larvae then continue to feed and grow in the plankton for about another 15 days before settlement and metamorphosis [49]. Members of this species can often be found in high numbers in shallow coastal waters, where they are important suspension-feeders [5], [48].

Substantial salinity fluctuations due to heavy rains are common in the tidal pools, intertidal areas, and shallow coastal areas inhabited by adults of *C. peruviana*. However, though *C. peruviana* is such an important member of Chilean coastal communities, there is little information on the responses and tolerance of this species to changes in salinity. Though some work exists on the tolerance of particular life history stages of *C. peruviana* to low salinity [7], we know little about the behavioral responses of adults or larvae to such stress. Additionally, although varying tolerance to salinity stress has been well documented for adults of many invertebrate species, there is less comprehensive information on the physiological mechanisms mediating poor performance in their sensitive larval stages.

In the present study we evaluated the impact of low salinity stress on the behavior of adults and of newly hatched veliger larvae of *C. peruviana.* In particular, we examined the ability of adults to isolate themselves from the external environment when faced with low salinity conditions, and investigated the effect of reduced salinity on the vertical distribution of veliger larvae. We also evaluated the impact of reduced salinity on key aspects of larval physiology and velum function. These experiments should help to identify the potential impact of global change on shallow-water marine animals, particularly those producing larvae that may be routinely exposed to altered salinities due to increased thawing caused by increased environmental temperatures, and to increases in local rainfall.

## Materials and Methods

Approximately 100 adults of the gastropod *Crepipatella peruviana* (35–45 mm SL) were obtained from Pelluco beach, Puerto Montt (41°28′S; 72°56′W) and transported to the laboratory. All individuals were immediately placed in a large aquarium with filtered seawater (0.5 µm), at 14°C and a salinity of 32, with constant aeration and daily feeding with the microalgae *Isochrysis galbana*. In order to avoid any genetic or maternal effects, thousands of newly-hatched veligers were subsequently collected from several different hatches and mixed before use in our experiments.

### Salinity threshold for adult isolation of brood chamber

Adults of *Crepipatella peruviana* were kept in an aquarium with an initial salinity of 32, equivalent to the salinity of the seawater from which specimens were collected. To determine whether the adults can seal themselves off from declining external salinity, and at what salinity this behavior is initiated, salinity in the experimental aquarium was lowered by 3–4 every hour by adding distilled water, until reaching a final salinity of 15. During this process, the water in the aquarium was constantly agitated to create a uniform salinity throughout the aquarium. Fluid from the female mantle cavity was sampled when the salinity in the aquarium water reached values of 32, 29, 25, 22, 19 and 15.

Ten different adults were removed from the aquarium at each sampling time, to obtain data on the salinity within the mantle cavity. In air, the specimens were carefully detached from the original substrate to which they were attached, always keeping the individuals inverted to prevent the loss of fluid retained in the mantle cavity. With 10 mL syringes, the fluid retained in the mantle cavity of each individual was extracted separately and deposited in a clean Eppendorf tube. Fluid from the mantle cavity of each adult was sampled inmediately after removing the individual from the aquarium. The conductivity of the fluid collected was then measured immediately using a portable multi-parametric device (Thermo Electron Corporation, Orion 5 Star) connected to a micro electrode of 2 mm diameter (Microelectrodes). Using appropriate computer software (SeaWater), the water conductivity measurements were transformed into salinity readings. Simultaneously, we recorded the salinity in the aquarium from which each individual had been removed. This information allowed us to determine the salinity at which females of this species first isolated themselves from the external environment.

### Salinity of fluid retained in the mantle chamber through female isolation

Adults were maintained in an aquarium with a salinity of 32. Gradually, the salinity was reduced to 15, to provoke females into isolating the mantle cavity from the external environment. To determine how long females were able to keep their mantle cavities isolated from the outside environment when exposed to hyposaline conditions for many hours, 10 different individuals were subsequently removed from the aquarium every 2 hours (for a total of 10 hours) and carefully detached from the substrate in air. Each individual was carefully maintained in an inverted position to prevent fluid loss from the mantle cavity. After removing the individual, samples of the pallial fluid were obtained from each individual with a 10 mL syringe, and put into separate Eppendorf tubes. Immediately, the salinities were determined for each sample, using a micro electrode 2 mm of diameter (Microelectrodes) as described above.

### Effect of salinity discontinuities on vertical distribution of *C. peruviana* early veligers

These experiments were conducted using glass tubes of 40 cm in length, 2 cm internal diameter and 0.2 cm thick to determine the impact of salinity gradients on larval distribution. The discontinuous salinities were obtained by first adding high salinity seawater (32, control) to the middle of each column, where a mark was made on the glass tube. We then slowly added seawater of a lower salinity to fill the rest of the column. In all of these experiments, the salinity in the bottom half of the column was always 32. Upper salinities were either 25, 20 or 15. By monitoring the salinity every 12 h for 48 h before conducting any actual experiments, we found that a strong and stable salinity discontinuity between the 2 different salinities was maintained in the tubes for at least 48 hours. Each salinity treatment had 6 replicates.

All salinity measurements were carried out a microelectrode that was 2 mm in diameter (Microelectrodes). Salinity levels were recorded at 2 cm intervals over the entire length of each glass tube. In the middle of the tube, we recorded a very well established mixing zone or interface of approximately 6 cm between the two layers. This mixing area was marked on the outside of each tube, 3 cm upwards and 3 cm downwards with respect to the initial mark (in the middle of the tube) made after having added the high salinity seawater (32) [46]. Thus, each experimental tube contained three main areas: a lower region of high salinity (1), an area of mixed salinity or discontinuity in the middle of the tube (2) and the upper region of the column with low salinity water (3). We also used 3 control tubes, which were filled only with high salinity water (32), and used to identify the larval vertical distribution at control salinity in the absence of any salinity gradient.

At the start of each experiment, 100 newly hatched veligers of *C. peruviana* were carefully deposited into the lower region of the tube containing high salinity (32) water, using a glass pipette. Inmediately after adding the veligers to the tubes, we used the microelectrode to be sure that the discontinuous salinities in each treatment were maintained. One hour after the veligers were placed in each tube, we recorded the number of veligers present every 5 (±1.5) cm in each salinity section along the entire water column. To identify the position of each larva, we used a standard magnifying glass. Immediately after determining the larval positions, the salinity throughout the water column was again checked using a microelectrode. The data obtained allowed us to determine the proportional distribution of veligers in the different discontinuous salinities for each one of the different experimental columns (32/32, 32/25, 32/20 and 32/15).

### Salinity and mortality (LC50) of *C. peruviana* veligers

Aquaria of 200 mL were prepared with 4 different levels of salinity (32 (control), 25, 20 and 15) by mixing filtered seawater (0.5 microns) with distilled water. Three replicates were used for each experimental salinity level. In each aquarium, 100 newly hatched veliger larvae (350 µm in SL) of *C. peruviana* were added. The larval veligers were fed by adding the microalgae *Isochrysis galbana* (80,000 cells mL^−1^). The water in each aquarium was changed daily, and live individuals were counted while the dead ones were counted and removed. Veligers without detectable velar ciliary movement were considered to be dead [51], [52]. Larvae in the aquaria were monitored until 50% of the larvae died.

### Impact of salinity on the velar activity and swimming velocity of the *C. peruviana* veligers

200 mL aquaria were filled with filtered seawater (0.5 µm) using the following levels of salinity: 32 (control), 25, 20 and 15. Each experimental salinity level had 4 experimental replicates. In each aquarium, 50 newly hatched veligers (350 µm SL) of *C. peruviana* were added; they were then maintained under those conditions for 6 h. After that time, to quantify the percentage of veligers with extended velar lobes in each aquarium, we observed all larvae using a stereomicroscope Leica model M80 at 100X magnification. The area of velar lobes (mm^2^) was then determined for each of those individuals using an inverted microscope (100X magnification) Olympus CK2, which was fitted with a video camera (Pulnix) and a digital recorder (Dahua technology). From recorded videos, photos of veligers were obtained whenever they were facing their velum to the observer. Using an image processor (Scion image pro), the edges of both lobes of the velum were traced and measured, and the velar surface area (mm^2^) was determined [53].

Larval swimming velocity was estimated for veligers exposed to the same treatments of salinity described previously. From the different levels of salinity, 10 veliger larvae from each experimental aquarium were removed and deposited on a glass depression slide with filtered seawater (0.5 µm). Using a Leica EZ4 magnifying glass equipped with a video camera (Pulnix) that was connected to a digital video recorder (Dahua technology) and a monitor (Sony), the movements of each larva from each treatment were recorded for one minute. In parallel, we recorded a graduated slide at the same magnification. Subsequently, using digital videos and the graduated slide, we determined the distance traveled by each veliger during the one-minute observation period. The time taken to travel a given distance was obtained by using the recording velocity (frames per time) as a reference. Distance traveled by veligers was measured in straight lines but over short distances, which reduced the problem of any non-linear movements made by the veligers. With this information, we were able to estimate the average displacement velocity (mm seg^−1^) for each veliger.

### Salinity and physiological performance of *C. peruviana* veligers: oxygen consumption rate (OCR) and clearance rate (CR)

During the OCR determinations, individual 10 mL syringes were filled with filtered seawater (0.5 µm). When necessary, we added distilled water to achieve the required levels of salinity: 32 (control), 25, 20 and 15. Six replicates were prepared for each salinity treatment. Each hermetically sealed syringe was filled with 4 mL of water at a particular salinity; the water had previously been saturated with oxygen by air continuous bubbling. Fifty newly hatched veligers (350 µm SL) of *C. peruviana* were then added to each experimental syringe, and maintained in the different salinity conditions for 6 h. The syringes were maintained floating in a thermostated bath at a constant temperature of 14°C. For OCR quantifications a Microx TX oxygen sensor was introduced through the tip of each syringe; oxygen concentration was measured at time 0 and after 6 h, which allowed us to estimate the mean oxygen consumption rates per individual for each syringe.

For CR quantification we used the same low salinity treatments previously described. The quantifications were made in triplicate for each salinity level, and at a constant temperature of 14°C, with 10 recently-hatched veligers of *C. peruviana* per syringe. In parallel, 3 syringes were used as controls, with the same conditions of the experimental syringes but without veligers. This allowed us to discount the possible impact of sedimentation and/or cell division in the experimental syringes. Initially, each experimental syringe had a concentration of 80,000 cells mL^−1^ of the microalgae *Isochrysis galbana*. After 6 hours, the remaining microalgal concentration in each syringe was quantified using an electronic particle counter (Beckman Coulter Z2). The mean CR per individual was then estimated for the larvae in each syringe following the methodology of Coughlan [54].

### Statistical analysis

When normality and homogeneity of variance were verified for our data, we used one-way ANOVA to determine the threshold isolation salinity for adult females and also for recording changes in the salinity of the fluid retained in the maternal mantle cavity during isolation from the external environment. We also used one-way ANOVA to determine the effects of exposure to low salinity on larval mortality (LC50), swimming velocity, velum area and percentage of veligers with an extended velum. The same statistical analysis was used to identify possible differences in the OCR and CR among veligers from the different treatments. By means of a two-way ANOVA we tested the differences between treatment and within the same treatment in the distribution of veligers throughout the water column. When significant differences between treatments were identified, *a posteriori* Tukey test was used to determine where the differences lay. In all analyzes, we used a significance level of 0.05 to determine whether differences were statistically significant or not [55].

### Ethics statement

The research was performed in an open access marine area and no specific permissions are required to extract resources from these locations. Also, species involved in this research is not endangered or protected.

## Results

### Salinity threshold for female mantle cavity isolation

**Figure 1:**
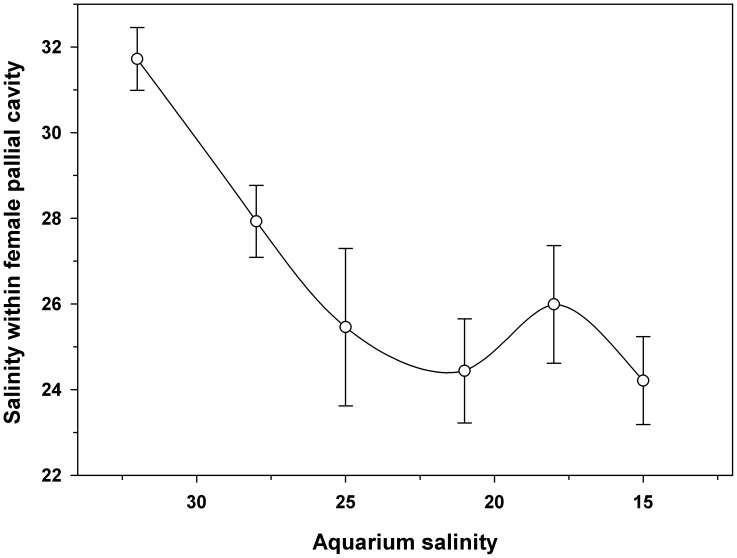
*Crepipatella peruviana:* Effects of reduced environmental salinity on isolation of the female pallial cavity. Each point represents means and SD (total n = 60, with 10 replicates per salinity value).

For females of *C. peruviana* maintained in aquaria with salinities between 32 and 25, mantle cavity salinities matched those of the surrounding seawater (Figure 1). However, even after the surrounding salinity declined to 15, the water retained in the mantle cavity never fell below 25 (Figure 1, one-way ANOVA, F_(5,60) = _20.3; p = 0.002), indicating that the adults had effectively isolated themselves from the outside environment at that level of environmental salinity.

### Salinity constancy of the fluid retained in the mantle cavity during female isolation

**Figure 2:**
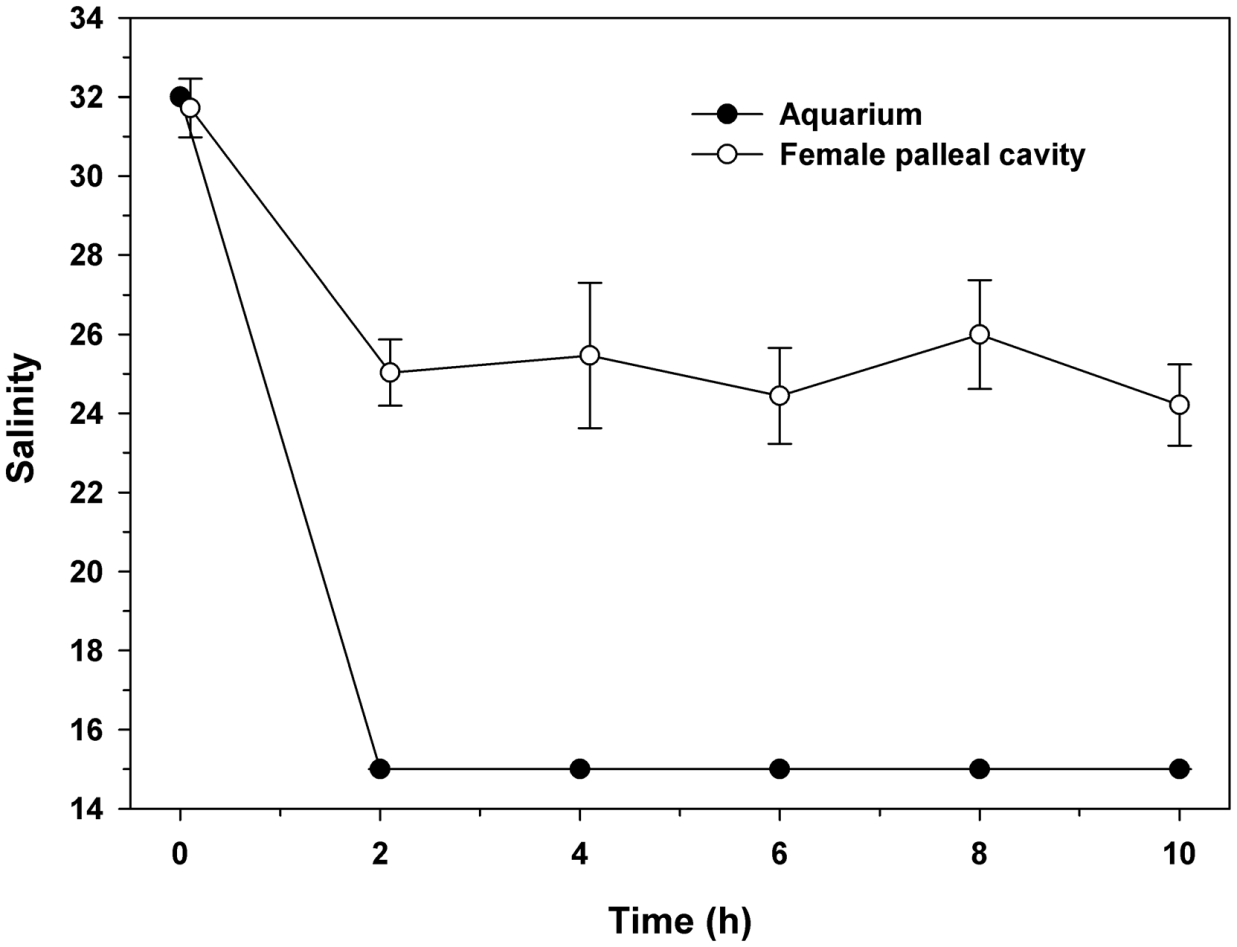
*Crepipatella peruviana*: Ability of adult females to maintain elevated salinity in the pallial cavity under conditions of low external salinity. Salinity was reduced from 32 to 15 at hour 2, as indicated. Each point represents the mean and SD (total n = 60, with 10 replicates for each time point).

When the salinity of the aquarium was maintained at 15, the maternal mantle fluid salinity remained constant at 25, with no significant variations throughout the 10 h experiment (Figure 2, one-way ANOVA, F_(5,60) = _0.58; p = 0.547).

### Vertical distribution of early veligers of *C. peruviana* in a water column with discontinuous salinities

**Figure 3:**
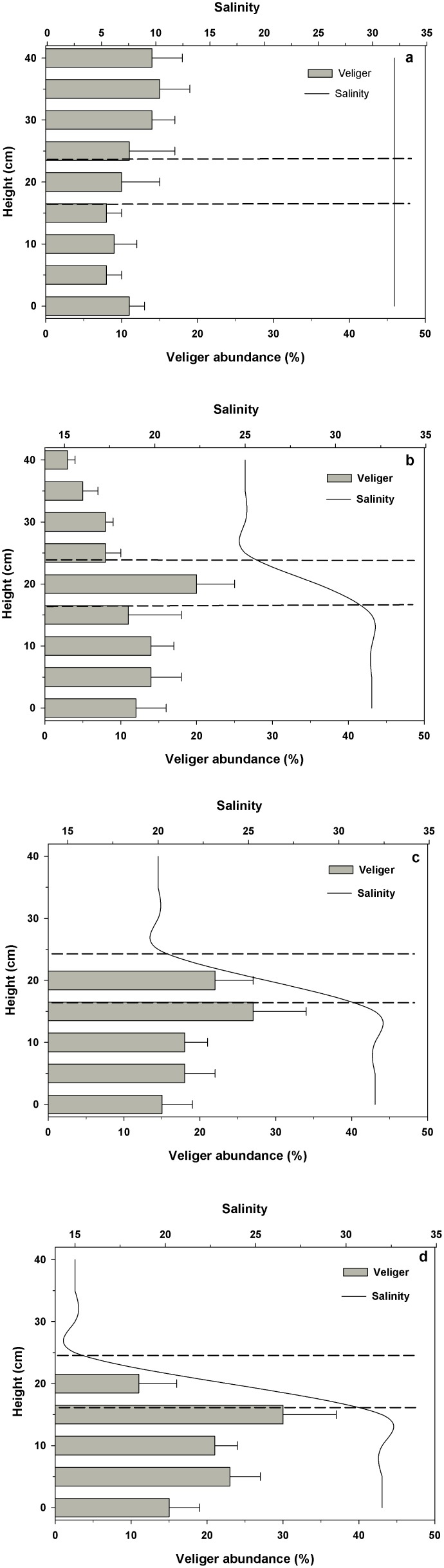
*Crepipatella peruviana:* The influence of salinity discontinuities on the vertical distribution of veliger larvae in a vertical water column. a: Control treatment: continuous salinity throughout at 32. b: discontinuous salinities of 32 in the lower half and 25 in the upper half of the column. c: discontinuous salinities of 32 and 20. d: discontinuous salinities of 32 and 15. The area between both horizontal dashed lines indicates the mixing zone or discontinuity between the two salinities. Each bar represents the means and SD of 6 replicates (n total = 24 columns and 100 veligers per columns).

Veliger larvae showed significantly different vertical distributions when exposed to salinity discontinuities (32/25, 32/20 and 32/15) (Two-way ANOVA, F_(6,24) = _13.6; p = 0.00001), compared to the larval distributions seen in the control treatment (32/32) (Figure 3, table 1). Veligers in the control treatment were distributed throughout the water column (Figure 3a), whereas in the other salinity treatments larvae remained below the salinity discontinuity, with a tendency to cluster near the discontinuity (Figure 3b, c, d). Within each treatment, significant differences (Two-way ANOVA, F_(2,36) = _84.07; p = 0.0001, table 1) were observed in the distribution of veligers within the 3 salinity regions defined in the water column.

**Table 1:** Results of a Two-way ANOVA performed on data from the experiment measuring the vertical distributions of *C. peruviana* veligers in environments of discontinuous salinity (32/32 (control), 32/25, 32/20, 32/15).

Source	Degrees of Freedom	MS	F	P-value
Intercept	1	38612.25	751.3735	0.00001
Treatment (salinity)	3	4.10	0.0798	0.97034
Vertical Distribution within the column	2	4329.75	84.0795	0.00001
Treatment × Vertical distribution	6	699.27	13.6074	0.00001
Error	24	51.39		

### Effect of salinity on *C. fecunda* veliger mortality (LC50)

**Figure 4:**
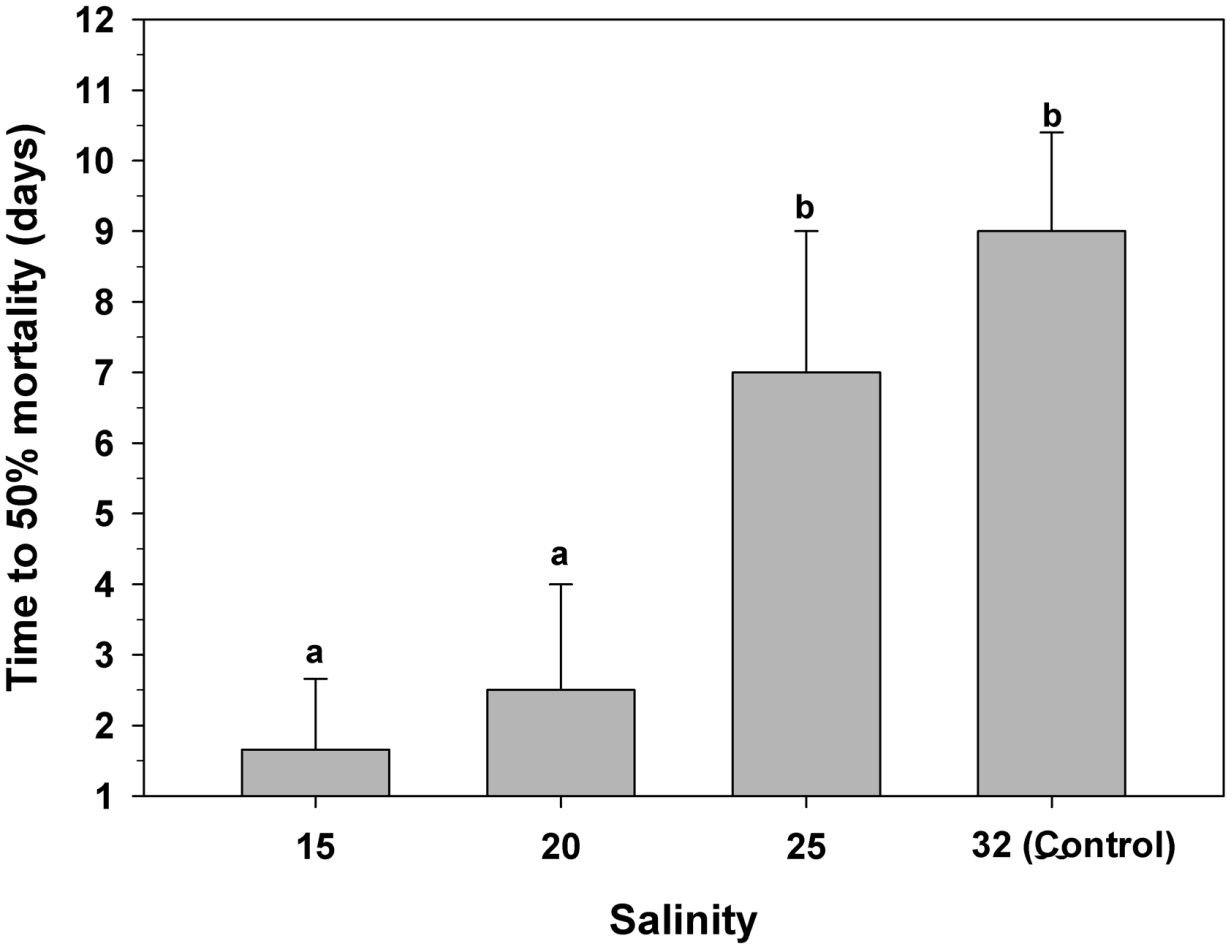
*Crepipatella peruviana:* Effect of low salinity on veliger mortality (LC50). Each bar represents means and SD (total n = 3 replicates per treatments, with 100 veligers per replicate). Different letters above the bars indicate significant differences (p<0.05).

In individuals held at the control salinity (32), 50% mortality was recorded after 9 (±1.2) days of pelagic life, and the same results were obtained for larvae exposed to a salinity of 25 (Figure 4). However, larvae exposed to salinities of 20 and 15 suffered significantly higher mortality (One-way ANOVA, F_(3,12) = _18.6; p = 0.001) compared to those in the control treatment. Mortalities of 50% occurred after only 2 (±1.4) and 1.6 (±1) days, respectively (Figure 4).

### Impact of salinity on larval velar activity and swimming velocity

**Figure 5:**
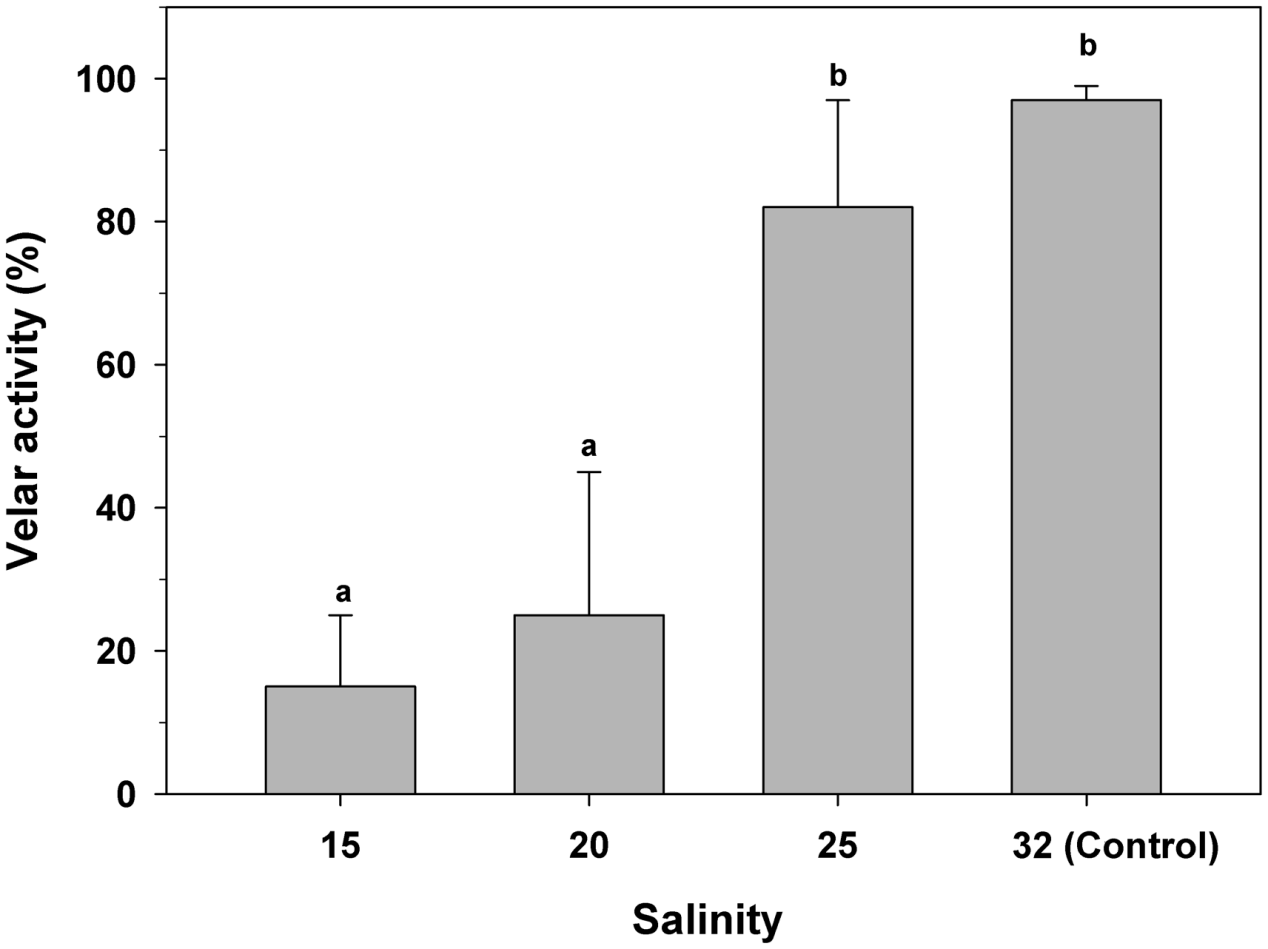
*Crepipatella peruviana:* Effect of low salinity on velar activity expressed as the percentage of veligers with extended velar lobes. Each bar represents the mean activity+one SD (total n = 4 replicates per treatments, with 50 veligers per replicate). Different letters above the bars indicate significant differences among means (p<0.05).

Veligers exposed to salinities of 20 and 15 showed significantly decreased velar activity (Figure 5, one-way ANOVA, F_(3,16) = _52.1; p = 0.0001). Only 25% (±15.3) and 15% (±10.1) of the larvae showed any velar activity at salinities of 20 and 15, respectively.

**Figure 6:**
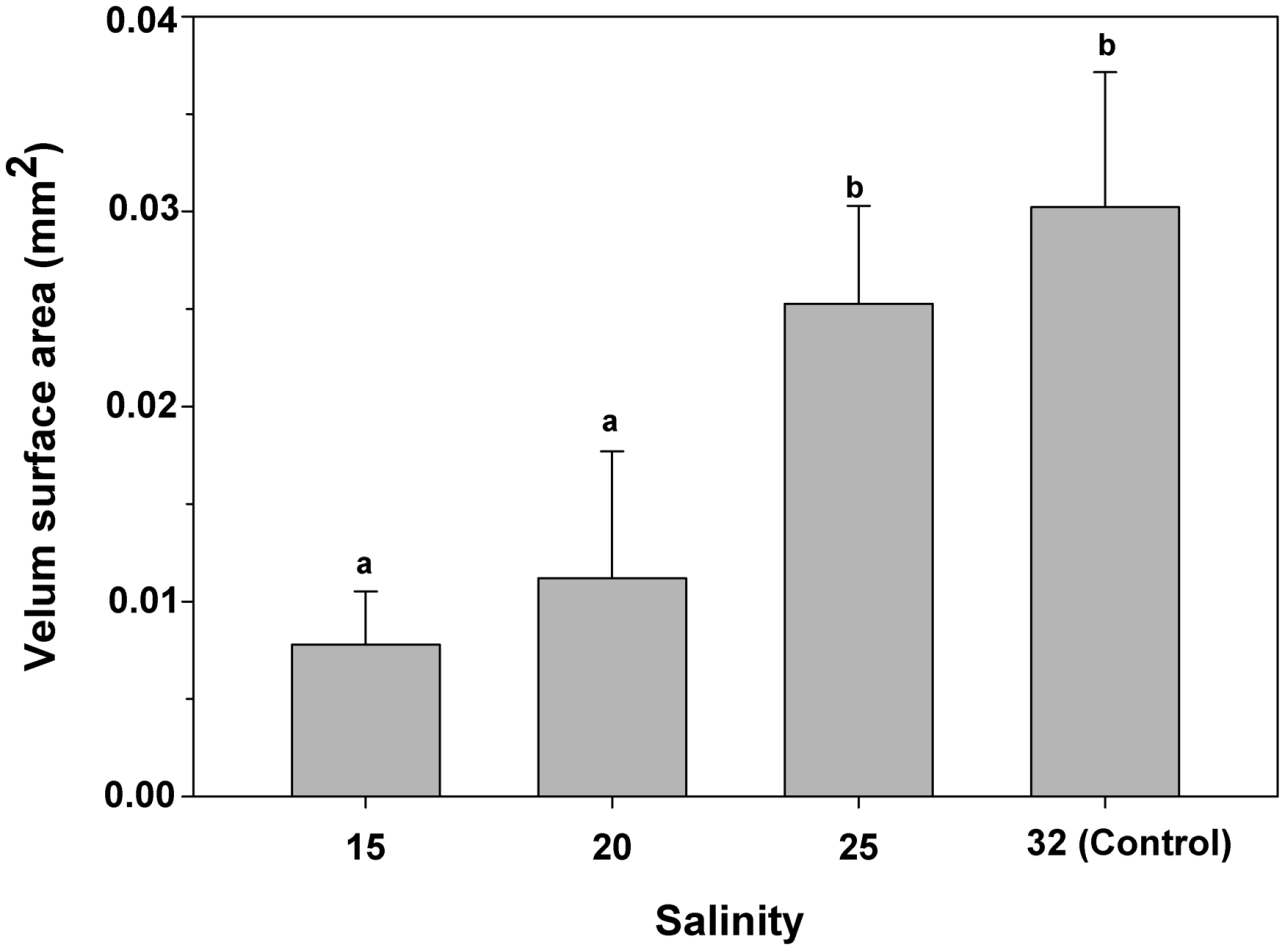
*Crepipatella peruviana:* Effect of reduced salinity on velar surface area. Each bar represents the means and SD (total n = 4 replicates per treatments, with 50 veligers per replicate). Different letters above the bars indicate significant differences (p<0.05).

The average velar surface area for larvae exposed to 25 was not significantly different from that of veligers at control salinity (32) (Figure 6). However, exposed velar surface area was significantly reduced for veligers exposed to salinities of 20 and 15, with a reduction of approximately 70% compared to control veligers (32) (Figure 6, one-way ANOVA, F_(3,160) = _63.6; p = 0.0002). Mean velum area was only 0.011 (±0.006) mm^2^ and 0.007 (±0.002) mm^2^ for larvae at salinities of 20 and 15, respectively.

**Figure 7:**
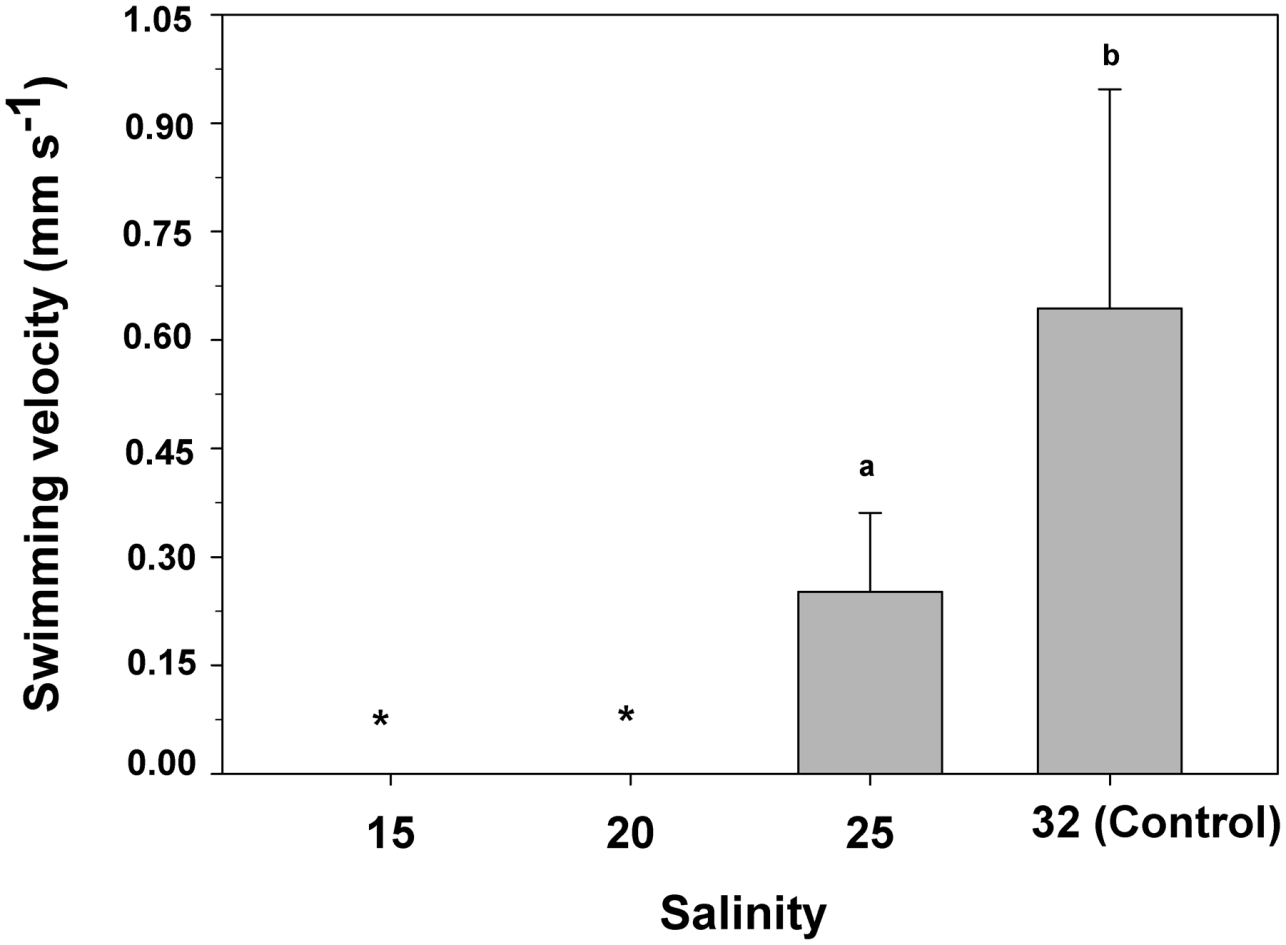
*Crepipatella peruviana:* Effect of ambient salinity on larval swimming velocity measured as the distance displaced by the veligers in a specified time. * Indicates absence of displacement. Each bar represents means and SD (total n = 4 replicates per treatment, with 10 veligers per replicate). Different letters above the bars indicate significant differences between means (p<0.05).

The veligers exposed to a salinity of 25 showed a significantly reduced (one-way ANOVA, F_(3,160) = _39,8; p = 0.001) mean swimming velocity, declining by about 60% from 0.64 (±0.30) mm s^−1^ (control, 32) to only 0.25 (±0.11) mm s^−1^ for larvae at 25 (Figure 7). Veligers exposed to salinities of 20 and 15 showed no displacement at all (Figure 7).

### Salinity and physiological performance of *C. peruviana* veligers: oxygen consumption rate (OCR) and clearance rate (CR)

**Figure 8:**
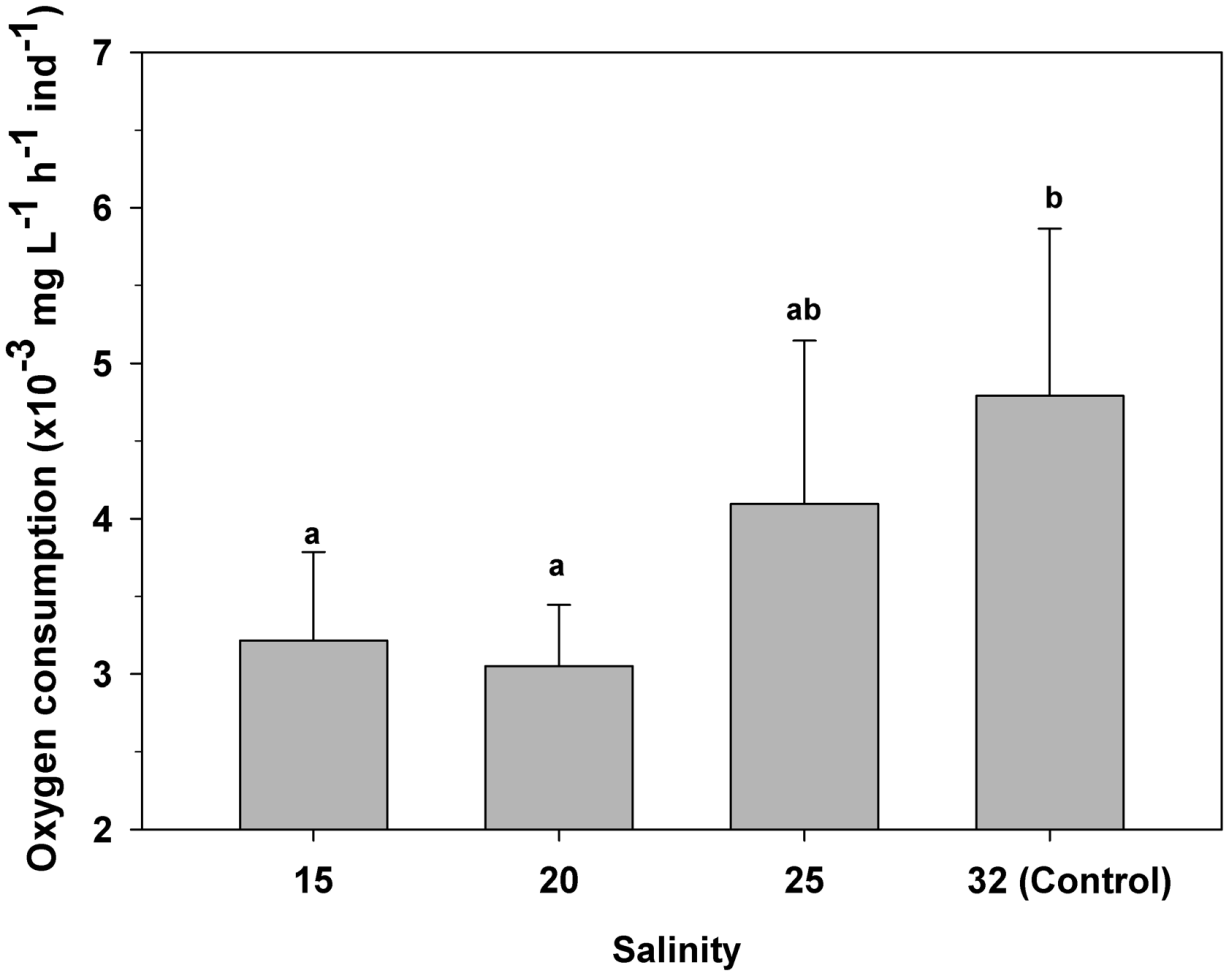
*Crepipatella peruviana:* Influence of reduced salinity on rates of oxygen consumption by young veligers. Each bar represents means and SD (total n = 6 replicates per treatments, with 50 veligers per replicate). Different letters above the bars indicate significant differences among means (p<0.05).

Exposing *C. peruviana* veligers to reduced salinity caused a significant reduction in OCR (one-way ANOVA, F_(3,24) = _52.1; p = 0.001). OCR was reduced by approximately 26% for larvae at salinities of both 20 and 15, with mean values 0.0030 (±0.0004) and 0.0032 (±0.0006) mg O_2_ h^−1^ ind^−1^, respectively (Figure 8). However, veligers exposed to 25 exhibited a mean OCR of 0.0040 (±0.0006) mg O_2_ h^−1^ ind^−1^, which was not significantly different from that of control larvae (0.0047 (±0.0006) mg O_2_ h^−1^ ind^−1^) (Figure 8).

**Figure 9:**
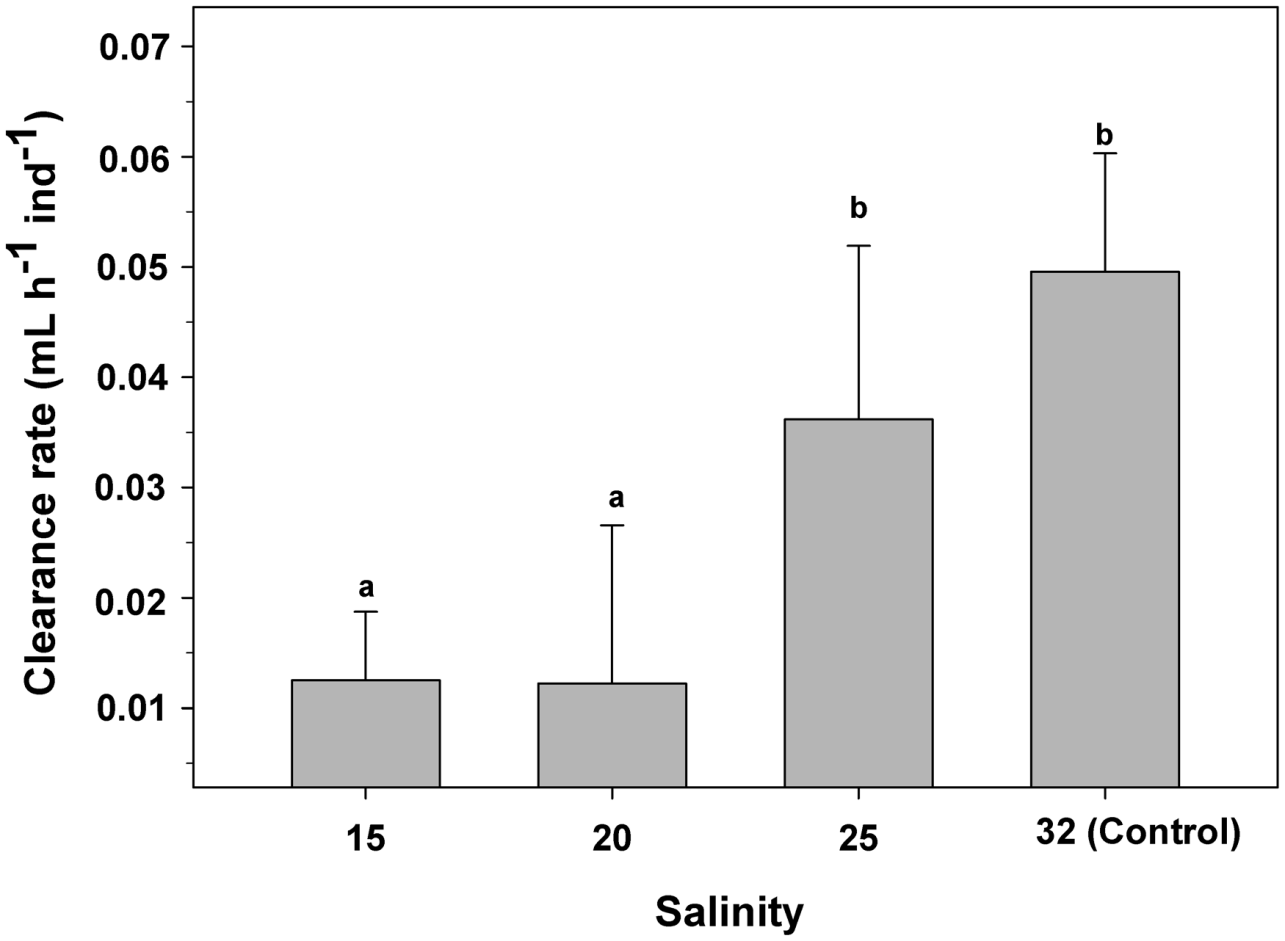
*Crepipatella peruviana:* Effect of salinity exposition on clearance rate of early veligers. Each bar represents means and SD (total n = 6 replicates per treatments, with 10 veligers per replicate). Different letters above the bars indicate significant differences (p<0.05).

The mean CR for control veligers (salinity of 32) (0.049±0.011 mL h^−1^ ind^−1^) was not significantly different from that of individuals exposed to 25 (0.036±0.016 mL h^−1^ ind^−1^). However, the mean CR of control larvae was significantly higher (one-way ANOVA, F_(3,24) = _29.7; p = 0.003) by approximately 300% than that of individuals exposed to salinities of 15 and 20 (Figure 9).

## Discussion

Salinity often plays a major role in determining the distribution of many marine organisms, primarily because of its effects on many physiological and behavioral processes [8], [15], [21], [36], [56], [57], [58], [59]. Some marine gastropods, when exposed to low environmental salinities, can isolate themselves from the outside environment by strong adhesion of the foot to the substrate [11], [32], [33], possibly protecting any brooded offspring in the process [11], [33], [60], [61]. In this research, females of *C. fecunda* began isolating themselves when environmental salinity was <24, a level slightly higher than that previously recorded for females of *C. dilatata* and the oyster *Ostrea chilensis* (salinity of 22.5, [33]). Environmental salinities can often be found at depths of 0 to 6 m in the waters of Gulf Reloncavi [62], [63] near Pelluco beach, where individuals of the present investigation were obtained. Additionally, salinities <24 are commonly found at Pelluco beach in tide pools during low tide and heavy rainfall; tidepool salinity may fall to as low as 15 (Person. obser.). We have also previously found *C. fecunda* larvae of hatching size (320–380 µm) in tide pools during the reproductive season [49]. Thus, both adults and larvae of *C. fecunda* could frequently be exposed to salinities ≤24 in the intertidal zone and in shallow subtidal areas.

The salinity of the seawater retained in the mantle cavity of *C. peruviana* adults remained near 24 for at least 10 h at reduced external salinity, even when adult individuals were exposed to external salinities as low as 15. This indicates that adults of *C. peruviana* can not only isolate the mantle cavity when external conditions of environmental salinity decline to stressful levels, but also that they can remain isolated for many hours until environmental conditions are again favorable for reopening and reconnecting with the external environment [11].

Although the *C. peruviana* mothers clearly have the ability to protect their brooded young for long periods of time in the face of declines in ambient salinity, the larvae obtain no such protection once they hatch, afterwards spending at least several weeks developing in the plankton. Although powerless to swim against a water current, many marine invertebrate larvae can change their vertical distribution in response to changes in biological and physical factors in the water column such as temperature, food and salinity (17, 26, 41, 46, 64, 65, 66, 67]. For example, discontinuous salinity levels can modify the vertical distribution of veligers of *Spisula solidissima, Mulinia lateralis* and *Rangia cuneata* [46]. Larvae can be found strongly aggregated in the water column, preferentially in the area of higher salinity or in the mixing zone (intermediate salinity) between different salinities, depending on their salinity stress tolerance [46]. Consistent with these findings, veligers of *C. peruviana* deliberately located themselves in the higher salinity (32, control) water when presented with a salinity discontinuity, and a lesser proportion were found in the mixing zone between the two salinities in the water column.

On the other hand, when no high-salinity waters were available, our study shows that the survival of *C. peruviana* is significantly reduced at lower salinity. Salinities of 20 and 15 killed at least 50% of the veligers within just 2 days of exposure. An equivalent mortality for control veligers was not recorded until 10 days had passed. These results corroborate previous studies identifying a low tolerance of larval veligers of *C. fecunda* to low salinity events, particularly when compared with those of *Crepidula fornicata* and *Crepidula onyx* [7].

The larval swimming organ, the velum, was negatively affected by low ambient salinity. In the present investigation, control veligers showed a velum area of 0.03 (±0.006) mm^2^, which coincides with previous information for newly hatched veligers of *C. fecunda* (0.04 mm^2^, [68]). However, in those veligers which had been exposed to salinities of 15 to 20, velar surface area significantly decreased by approximately 65%, in comparison to that of control veligers. This situation should affect larval activities such as swimming velocity, food capture ability, and respiration rates (68, 69, 70, 71, 72, 73, 74], considering the active participation of the velum in all of those processes. This situation can be exemplified very well by the lack of movement recorded in this investigation, when veligers were exposed to salinities of 15 and 10.

The ability of larval gastropods to feed is directly related to the size of the filtering area of the velum [68], [74], [75], which results from a relationship between the velum perimeter and the length of the velar cilia composing the opposing-band feeding system [68], [76]. The clearance rate recorded for the control veligers of *C. peruviana* coincides with those recorded previously by Chaparro et al. [68]. By contrast, the veliger larvae of *C. peruviana* that were exposed to low levels of salinity (20 and 15) showed clearance rates that were approximately 70% lower than those of larvae in the control treatment. A similar result has been recorded for the larvae of *Crepidula fornicata*, which showed a decrease of about 75% in clearance rate when they were exposed to salinities of 15 and 10 [7].

Exposure to stressful low salinities reduced the OCR of veliger larvae of *C. peruviana* by 40% at salinities of 20 and 15, compared to the values recorded in the control treatment. Negative impacts of low salinities on OCRs have also been recorded for larvae of various invertebrate species [37], [42], [43], [44], [45], [77], [78], [79]. Because the gastropod velum has been described as a major respiratory organ for gastropod larvae [72], the decrease in oxygen consumption by *C. peruviana* larvae would be closely linked to the lack of activity and decreased size of the velum in stressed veligers, considering that a smaller velum implies a smaller surface area available for gas exchange [72].

In summary, adults of *C. peruviana* isolate their mantle cavity from the external environment in response to a low external salinity of about 24. Thus, adults and brooded embryos are protected from exposure to reduced external salinity in the short term, although they run the risk that a prolonged period of isolation can be detrimental to the incubated embryos due to various negative changes that are generated in the mantle fluid over time when it is sealed off from external environment [33], [80]. But this female behavior clearly prevents the brooded embryonic stages from ever being exposed to low salinity stress, even when the external environment has low salinity for prolonged periods. Although newly hatched *C. peruviana* veligers may be able to avoid low-salinity exposure if there are high-salinity regions available in the vertical water column, they are otherwise very vulnerable to the physiological stresses imposed by reduced salinity. The negative impact on larvae is immediate, affecting a number of important physiological processes and larval behaviors. Future studies should determine the possible impact of such adverse environmental conditions on subsequent larval development and also consider the potential latent impacts [81] that such exposure might have on juveniles and adults of this species.
